# Interdisciplinary Sport Research Can Better Predict Competition Performance, Identify Individual Differences, and Quantify Task Representation

**DOI:** 10.3389/fspor.2020.00014

**Published:** 2020-02-27

**Authors:** Ben Piggott, Sean Müller, Paola Chivers, Ashley Cripps, Gerard Hoyne

**Affiliations:** ^1^School of Health Sciences, University of Notre Dame, Fremantle, WA, Australia; ^2^Discipline of Exercise Science, Murdoch University, Murdoch, WA, Australia; ^3^Institute for Health Research, University of Notre Dame, Fremantle, WA, Australia; ^4^Exercise Medicine Research Institute and School of Medical and Health Sciences, Edith Cowan University, Joondalup, WA, Australia

**Keywords:** interdisciplinary research, individual differences, representative task design, sports science, Australian football

## Abstract

Sport performance consists of interacting individual, task and environmental constraints, but research has used a monodisciplinary, rather than an interdisciplinary approach to understand performance. This study used Australian football (AF) as the exemplar sport to investigate the value of an interdisciplinary approach to understand sport performance. Through this, it was also possible to quantify individual differences and representative task design. Fifty-nine semi-professional Australian footballers participated. Based upon accessibility, combinations of these players completed physiological (3 × 1 km trial) and perceptual-cognitive-motor (small-sided game, SSG) tests, with coach rating of psychological skill (mental toughness coach, MTC). Univariate monodisciplinary models indicated that all tests predicted disposal efficiency; 3 × 1 km trial (*p* = 0.047), SSG (*p* = 0.001), and MTC (*p* = 0.035), but only the SSG predicted coaches' vote (*p* = 0.003). A multivariate interdisciplinary model indicated that SSG and MTC tests predicted disposal efficiency with a better model fit than the corresponding univariate model. The interdisciplinary model formulated an equation that could identify individual differences in disposal efficiency. In addition, the interdisciplinary model showed that the higher representative SSG test contributed a greater magnitude to the prediction of competition performance, than the lower representative MTC rating. Overall, this study demonstrates that a more comprehensive understanding of sport performance, individual differences, and representative tasks, can be obtained through an interdisciplinary approach.

## Introduction

Sport performance has been frequently studied based upon physical or physiological components such as agility and aerobic capacity (Cardinale, 2017). Coaches and applied scientists, however, believe that competition performance involves an interaction between physiological, psychological, and perceptual-cognitive-motor components (Cardinale, 2017; Zaichkowsky and Peterson, 2018; Bonney et al., 2019a). For example, mid-field players in Australian football (AF) and soccer run approximately 10 kilometers per game, whilst attempting to make accurate skill decisions under high-pressure situations. Accordingly, several recent books (e.g., Ericsson et al., 2018; Zaichkowsky and Peterson, 2018) and scientific publications (e.g., Glazier, 2015; Cardinale, 2017) have discussed the importance of psychological and perceptual-cognitive-motor components, which need to be considered with physiological components, to provide a more comprehensive understanding of sport performance. This presents an opportunity to extend mechanistic (or theoretical) understanding of sport performance from an interdisciplinary perspective, which has applied implications in terms of identification of strengths and deficiencies of individual athletes for remediation.

Recent reviews of the sports science literature have found that interdisciplinary research continues to be scarce (Buekers et al., 2016; Piggott et al., 2019c). There are several possible reasons for why this is the case. First, sports science researchers may not be aware of suitable theoretical frameworks that can underpin interdisciplinary research questions, experimental design, and analyses (Buekers et al., 2016). Second, interdisciplinary research is highly dependent upon the organization of a team of scientists who have less preference upon a monodisciplinary approach to understand sport performance (Buekers et al., 2016). Third, interdisciplinary research can require greater resources and participant time, which may limit sport performance to be investigated from a monodisciplinary perspective (Buekers et al., 2016). Nonetheless, Buekers et al. (2016) and Piggott et al. (2019c) have presented solutions to these barriers in the form of theoretical frameworks to guide experiment and test design. These authors also mentioned the incentive for researchers to conduct interdisciplinary research, which is its capacity to provide a comprehensive understanding of athletic performance.

A useful framework to underpin interdisciplinary research is constraints theory (Higgins, 1977; Newell, 1986), because it takes into consideration interacting variables that can shape achievement of the motor skill goal. Constraints include interacting components related to the individual, task and immediate environment that can guide achievement of the motor skill goal (Higgins, 1977; Newell, 1986). An example of constraints is demonstrated in the last minute of a rugby game, when the team in possession of the ball is behind by four points (environment). The halfback (individual) of the losing team throws a pass to the winger, the fastest player on their team who has open space in front of him. This gives the halfback an opportunity to score a try (task), whilst avoiding being tackled by an opponent(s), which would result in the team winning the game. In relation to individual constraints; physiological, psychological, and perceptual-cognitive-motor components interact with the environment and task constraints to attempt successful achievement of the motor skill goal. Therefore, constraints theory predicts that interacting variables, aligned with an interdisciplinary approach, are crucial to comprehensively understand performance in sport.

Underpinned by constraints theory, it is possible to develop research questions, experimental design, and analyses to further understanding of sport performance. AF can be chosen as an exemplar sport because it includes interacting physiological, psychological, and perceptual-cognitive-motor individual constraints (Bonney et al., 2019a). A key task constraint of AF competition is effective disposal of the ball to a teammate without being tackled or the pass being intercepted. This refers to the skill (or task) goal in competition, which can be measured using disposal efficiency (Piggott et al., 2019b). Disposal efficiency is defined as the percentage of disposals that are effective or hit their intended target and is one of the match statistics provided by a commercial analytics company (Champion Data, South Bank, Australia). Passing the ball in AF occurs under a highly dynamic environmental constraint, because there are multiple players in close proximity to the ball carrier who can tackle from any direction. Therefore, by systematically integrating constraints on the individual, it is possible to determine, as predicted by constraints theory, whether disposal efficiency can be better predicted through a monodisciplinary or interdisciplinary approach. This contributes to theoretical understanding through interacting constraints that may influence sport performance.

Although constraints theory predicts performance based upon the individual, sports science researchers have focused upon group designs, rather than sub-groupings or analyses that can reveal individual differences (Piggott et al., 2019c). The latter analyses have important practical implications, as coaches are interested in the strengths, deficiencies, and development of individual athletes, so that interventions can be tailored to improve individual athlete competition performance. For example, underpinned by constraints theory, Chow et al. (2008) reported individual differences in hip, knee, and ankle joint coordination when novices learned to chip kick a soccer ball to targets of varying distance and size. This study demonstrated that more than one coordination pattern can be used to learn a skill. In another example, Piggott et al. (2019b) used a sub-grouping analysis relative to games played and position of play within skilled Australian footballers. They reported superior decision-making in SSG's relative to increased exposure to competition and playing in the mid-field position. This study demonstrated that task experience and position of play can influence decision-making capability. Accordingly, it has been suggested that in order for sport performance research to best guide athlete preparation, individual, or sub-groups analyses are necessary to better elucidate interaction with task and environment constraints (Woods et al., 2019).

Design of tests or selection of measurement instruments is also an important consideration for interdisciplinary research to best capture interacting constraints of sport performance. This refers to representative task design, which includes properties of the test or measurement instrument and their generalization to the intended context (Araújo and Davids, 2015), which in sport is the competition setting. It has been argued that properties (constraints) of a test such as perceptual information and action responses need to be sampled from the competition setting to be included in design of the test (Pinder et al., 2011b). For example, a SSG would be considered high in task representation because perceptual and action components are closely related to competition performance, whilst coach rating of athlete performance involves none of these components, so it would be considered low in task representation. In a related manner, the literature indicates that practice drills in AF allow the ball carrier to retain possession of the ball for considerably longer prior to a decision to dispose, than occurs in competition (Woods et al., 2019). The implication of this finding is that coaches need to design practice tasks where athletes make accurate decisions, where ball possession time is like competition. Interdisciplinary research including physiological, psychological, and perceptual-cognitive-motor component tests can provide an indication of what best predicts a measure of match performance such as disposal efficiency. Through this, it is possible to obtain an understanding of which interacting test(s) or measure(s) represents sport performance. This will help coaches and sports scientists select representative talent identification and test batteries for athlete evaluation and training.

This study employed both monodisciplinary and interdisciplinary approaches using AF as an exemplar sport. We used three sports science sub-discipline measures to examine their relationships to two match performance statistics in disposal efficiency and coaches' vote. The purpose of this study were to determine: (a) whether an interdisciplinary approach provides a more comprehensive understanding of athlete match performance than a monodisciplinary approach, (b) whether an interdisciplinary approach could provide an equation to quantify individual player performance profiles, and (c) representativeness of measures in terms of their capability to predict match performance. It was hypothesized that: (i) an interdisciplinary approach would provide a better model fit in predicting a measure of match performance (disposal efficiency), in comparison to a monodisciplinary approach, (ii) individual player profiles would provide enhanced insight into sport performance, which is less evident in group descriptive analyses, and (iii) measures that closely represent competition would more accurately predict match performance.

## Materials and Methods

### Participants

A total of 59 male players (M_age_ = 21.27 ± 3.11 years, M_height_ = 186.79 ± 7.17 cm, M_bodyweight_ = 84.0 ± 9.13 kg) from a semi-professional AF league club were recruited for this study. All players were members of the senior playing squad and the inclusion criteria was that participants had to be free from injury at the time of testing. Human Research Ethics Committee (HREC) approval was received from the relevant university committee and participants provided written informed consent. This study was conducted in accordance with the Declaration of Helsinki.

### Materials and Procedures

The sport of AF requires a unique mix of physical, mental, technical, and decision-making skills (Young and Pryor, 2007). These skills can be classified into the following sub-disciplines of sport science according to Abernethy et al. (2013); exercise physiology, sports psychology and motor control. A test from each sub-discipline was selected and these were; (i) 3 × 1 km time trial (exercise physiology), (ii) mental toughness coach rating (sport psychology), and (iii) SSG test in AF (motor control). Justification for the selection of these tests is outlined below. The match performance measures used were disposal efficiency and coaches' vote. The three tests were all completed toward the end of the pre-season training period and before competition games began. Match performance measures were collated from a total of 20 home and away games over the 2017 competitive season.

### 3 × 1 km Time Trials

Literature reports that the running demands of AF are high (Coutts et al., 2010; Delaney et al., 2017). As a result, a primary focus of AF training is to develop players' capacity in this area (Delaney et al., 2017); a performance test that provides a measure of running ability was therefore used in this study. Traditionally, AF clubs have used a 3 km time trial in order to assess aerobic capacity (Gastin et al., 2013; Piggott et al., 2015). Recently, in an attempt to ensure that performance tests reflect the intermittent running requirements of the competition environment, an alternative 3 × 1-kilometer time trial test has been reported in the literature (Cripps et al., 2018). The repeated time trial test allows players to attain higher average running speeds (283 m/min ^−1^) than the more traditional 3 km time trial (252.8 m/min ^−1^) (Cripps et al., 2018). Furthermore, high intensity running has been linked to a player's capacity to win possession of the ball more frequently (Mooney et al., 2011). There is also an expectation from coaches that players can perform high intensity efforts interspersed with frequent short breaks on the interchange bench to recover.

The test required players to complete each 1-kilometer trial as quickly as possible running around a grassed oval. Each subsequent trial began 7:30 min after the start of the preceding trial. The total time taken by the player to complete the three trials represented the criterion measure. The 3 × 1 km time trial has acceptable test-retest reliability assessed using interclass correlation coefficients (ICC = 0.97, *p* < 0.01) and typical error measurements (4.36%) (Cripps et al., 2018).

### Mental Toughness Coach Rating

Mental toughness (MT) has been defined as a capacity for an individual to produce high levels of objective and subjective performance consistently despite everyday challenges, stressors and significant adversities (Gucciardi et al., 2015). There has been significant interest from researchers, practitioners and the general public in recent years regarding MT as a key factor of superior performance in a variety of domains including sport (Giles et al., 2018). The MT Index (MTI) developed by Gucciardi et al. (2015), is an eight item scale and an example item is; *I am able to execute appropriate skills or knowledge when challenged*. Recently, Piggott et al. (2019a) used a coach rating of the MTI and reported that this measure predicted performance in a high-pressure SSG. To assess MT in this study, a senior member of the club's coaching staff rated each players' level of MT using the MTI. This test has a low level of task representation because it is a survey and does not require a perceptual-motor response (Piggott et al., 2019c).

### Small-Sided Games

A recently developed SSG test in AF by Piggott et al. (2019b) was used to measure decision making and skill execution. The study reported that the test was highly reliable and was able to discriminate performance between semi-professional and amateur Australian footballers. In addition, the total score (summation of decision making and skill execution scores) significantly predicted disposal efficiency in competition performance (Piggott et al., 2019b). SSG's allow players greater opportunities to gain possession of the ball and display their skill level, as well as apply game strategy and tactics (Bonney et al., 2019b). The SSG test has a high level of representative task design as perceptual information that occurs in competition is presented to the participant and opportunity is provided to couple action to available perceptual information like the competition setting (Piggott et al., 2019c).

In the SSG tests, participants were divided into two teams of six attackers and five defenders. Attackers attempted to maintain possession of the ball in a 40 × 40 m grid using a hand pass or kick, whilst defenders tried to intercept, tackle, or spoil. Each skill group completed three sets each in attack and defense and each set was 3 min in duration. Prior to the commencement of the SSG's, participants completed a 15-min warm up which included running drills, dynamic stretching, basic kicking, and handball exercises. The test was umpired by a research assistant with AF knowledge. Each time an attacking player passed the ball by a kick or handball it was recorded as a trial. The decision making and motor skill execution scores for each disposal (trial), as well as both combined (total score) were coded as objective performance measures. A standard 25 Hz video camera (Panasonic SDR-H250, Australia) was used for recording so that trials could be coded post testing.

### Disposal Efficiency

A commercial statistical analytics company (Champion Data, South Bank, Australia) recorded each participants' disposal efficiency, which is the percentage of disposals (kicks or handballs) that were effective during each WAFL competition game. Champion Data is used extensively in AF and provides statistics for professional and semi-professional competitions with reported 99% accuracy (O'Shaughnessy, 2006). In addition, Champion Data statistics have been used in previous published research in AF (Mooney et al., 2011; Piggott et al., 2015).

### Coach Vote

Coaches' votes as a measure of match performance have been used in previous research (Mooney et al., 2011; Piggott et al., 2015). Mooney et al. (2011) used coaches' votes as a measure in their research to quantify the player's subjective performance, which encompasses both tactical and technical performance. For this study, each of five coaches, voted after every competition game, and each coach had 20 votes they could allocate across the participants who had played. The maximum votes an individual coach could give a participant was three votes. For example, if all five coaches gave a participant two votes each, then that participant would have recorded ten votes for that match.

### Data Analysis

Statistical analysis was performed using IBM SPSS version 25 (IBM SPSS Statistics for Windows. IBM Corp., Armonk, NY, USA) and Stata version 15 (StataCorp, College Station, Texas, USA). Data was checked for normality using the Shapiro Wilk's test so that the appropriate statistical tests could be employed. Alpha level was set at <0.05.

Hypothesis one was investigated using a series on univariate Generalized Estimating Equations (GEE). GEEs are an extension of general linear models, and are considered an appropriate approach for the analysis of correlated continuous and count data, and have the advantage of unbiased estimation of parameters (Ghisletta and Spini, 2004). In the GEE model, each sub-discipline performance test was the independent variable. For the first series of models, the match performance measure of disposal efficiency was the dependent variable and for the second series of models, coach vote was the match performance measure and dependent variable. Disposal efficiency employed a linear GEE model. For coaches' vote, a negative binomial link GEE model was used because of the over-dispersion of “zero” votes. The GEE model probability of not receiving a coaches' vote was graphically depicted using STATA (StataCorp. 2001). Each independent sub-discipline test was regarded as a monodisciplinary approach to quantify a component of individual constraints. Performance tests displaying significant prediction of the dependent variables in the univariate GEEs, were then integrated into a multivariate GEE model, which was regarded as an interdisciplinary approach. This is consistent with the literature, which has stated that integration of measures from different sub-disciplines using analyses such as stepwise regressions and prediction equations, can be classified as interdisciplinary (Freedson, 2009; Piggott et al., 2019c). To compare the fit of different GEE models, the Quasi Likelihood under Independence Model Criterion (QIC) was used where the model that obtains the smaller QIC has the better fit (Pan, 2001).

To address hypothesis two, the participants' mean disposal efficiency and coach vote from their match performance across the 2017 season was used. For these measures, participants were allocated into three groups depending on their performance and categorized as; high, medium and low performing. A panel of three experts who coached at semi-professional and professional levels agreed on the following cut-points for disposal efficiency: High (≥70%), Medium (60–70%) and Low (<60%), and coach vote: High (> 2 votes), Medium (≥ 1and ≤ 2 votes) and Low (<1 vote). Differences between categorized performance levels in sub-discipline tests was examined using ANOVA (or non-parametric Kruskal Wallis) with a Bonferroni *post hoc* comparison. In addition, Cohen's *d* effect sizes were calculated, with 0.2 considered small, 0.5 medium and 0.8 large effects (Cohen, 1998).

In regards to hypothesis three, using the estimates from the GEEs predictive equations, we examined a 10 percent change for high and low level representative tests and determined prediction relative to the dependent variable. In addition, the QIC was used to assess model fit for tests of both high and low representative task design.

The number of participants that completed the tests were as follows: 3 × 1 km time trial (*n* = 46), mental toughness coach rating (*n* = 59), and SSG AF test (*n* = 28). The final multivariate model assessed a total of 21 (M_age_ = 21.24 years, Age range: 18–29 years) players who completed all three performance tests. Not all players could complete all three tests due to player injury, load management, and training scheduling. In addition, injury restricted participants availability to participate in every match throughout the year and this is common in contact team sports. For the final multivariate model, the average number of matches played over the season per participant was 15.6, which is similar to 15.5 matches reported in previous research with a similar focus (Gabbett et al., 2011).

## Results

### Monodisciplinary and Interdisciplinary Approaches

At the univariate (monodisciplinary) level (Table 1), all three sub-discipline performance tests were significant predictors of match disposal efficiency; 3 × 1 km time trials (β = −0.07, *p* = 0.047), mental toughness coach (β = 0.37, *p* = 0.035), and SSG test (β = 12.51, *p* = 0.001). At the univariate (monodisciplinary) level, only SSG test was a significant predictor of coaches' vote (β = 1.19, *p* = 0.003) (Table 2).

**Table 1:** Univariate generalized estimating equations for prediction of disposal efficiency.

**Model**	**Parameter**	**Estimate (β)**	**SE**	**95% CI**	***p***
Model 1 (QIC 176,119)	Intercept	108.45	21.97	65.39 to 151.52	0.001
	3 × 1 km	−0.07	0.03	−0.13 to 0.01	0.047^*^
Model 2 (QIC 2234,463)	Intercept	48.06	7.91	33.56 to 63.57	0.001
	MTC	0.37	0.18	0.03 to 0.72	0.035^*^
Model 3 (QIC 110,014)	Intercept	−1.62	18.48	−37.85 to 34.61	0.930
	SSG Test Score	12.51	3.50	5.65 to 19.38	0.001^*^

* *Indicates a significant difference p < 0.05. SE, standard error; CI, confidence intervals; MTC, Mental Toughness Coach; QIC, Quasi Likelihood under Independence Model Criterion*.

**Table 2:** Univariate generalized estimating equations for prediction of coaches' vote.

**Model**	**Parameter**	**Estimate (β)**	**SE**	**95% CI**	***p*-value**
Model 1 (QIC 1141)	Intercept	1.78	2.68	−3.48 to 7.04	0.507
	3 × 1 km	−0.01	0.01	−0.01 to 0.01	0.185
Model 2 (QIC 1424)	Intercept	−3.05	0.98	−4.97 to −1.12	0.002
	MTC	0.03	0.02	−0.02 to 0.07	0.210
Model 3 (QIC 697)	Intercept	−8.07	2.18	−12.35 to −3.79	0.001
	SSG Test Score	1.19	0.40	0.39 to 1.99	0.003^*^

* *Indicates a significant difference p < 0.05. SE, standard error; CI, confidence intervals; MTC, Mental Toughness Coach; QIC, Quasi Likelihood under Independence Model Criterion*.

When the significant independent variables from the univariate disposal efficiency model were integrated into a multivariate (interdisciplinary) model (Table 3), both mental toughness coach (β = 0.37, *p* = 0.002) and SSG test (β = 12.34, *p* = 0.001) remained as significant predictors, whilst the 3 × 1 km test was not significant (β = −0.06, *p* = 0.077). A multivariate (interdisciplinary) model of coach vote was not required as only one performance test, the SSG test score, was found to be a significant predictor. The Goodness of Fit measures (QIC's) are reported for all GEE's within their respective models (Tables 1–3). For disposal efficiency, where a multivariate (interdisciplinary) model was developed (Table 3), the QIC value was lower than the QIC values for the univariate models, indicating a better model fit.

**Table 3:** Multivariate generalized estimating equations for prediction of disposal efficiency (*n* = 21).

**Parameter**	**Estimate (β)**	**SE**	**95% CI**	***p***
Intercept (QIC 84,155)	22.88	31.65	−39.15 to 84.92	0.470
3 × 1 km	−0.06	0.03	−0.13 to 0.01	0.077
MTC	0.37	0.12	0.13 to 0.61	0.002^*^
SSG Test Score	12.34	3.08	6.29 to 18.38	0.001^*^

* *Indicates a significant difference p < 0.05. SE, standard error; CI, confidence intervals; MTC, Mental Toughness Coach; QIC, Quasi Likelihood under Independence Model Criterion*.

The predictive equation resulting from the multivariate (interdisciplinary) model for disposal efficiency was:

*DE* = *22.88* + *(-0.06) x 3 x 1k time* + *0.37 x MTC score* + *12.34 x SSG test score*

As an example, if the mean score for each performance test was used, the equation would be:

= 22.88 + (−0.06) × 645.24 + 0.37 × 43.19 + 12.34 × 5.31

= 66%

**Figure 1:**
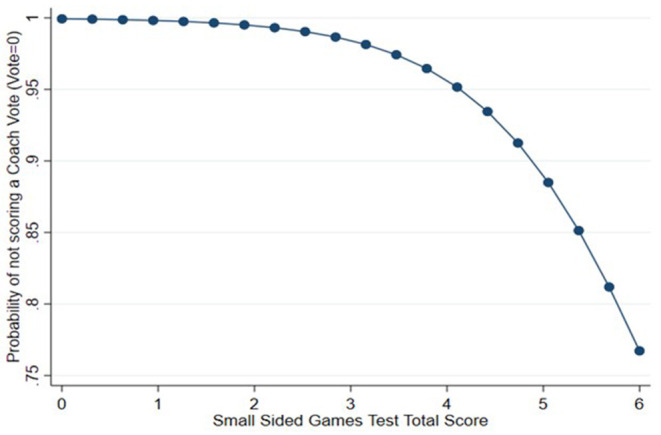
The probability of not scoring a coach vote in relation to small sided games test score.

For coach vote, as a negative binomial link model was used, this limits the ability to create a meaningful predictive equation as presented for disposal efficiency. However, the modeled probability of not receiving a coach vote in relation to a participant's SSG test score can be graphically depicted (Figure 1). It can be seen from Figure 1 that when a participant's SSG test score is approximately 3.5 or higher, then the likelihood of them not receiving a coach vote is substantially reduced (i.e. likely of receiving a coach vote is increased).

### Group and Individual Differences

**Figure 2:**
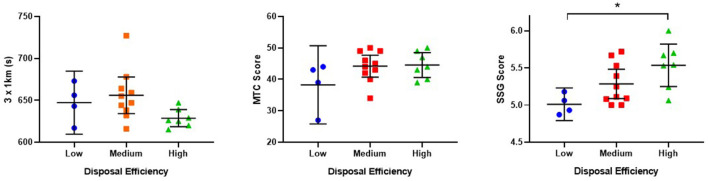
Mean and 95% CI scores for sub-discipline performance test scores for low, medium and high scoring groups for disposal efficiency. *Indicates significant difference *p* < 0.05 between sub-groups. MTC, Mental Toughness Coach; SSG, Small sided games.

**Figure 3:**
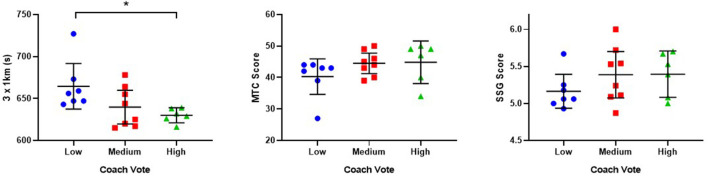
Mean and 95% CI scores for sub-discipline performance test scores for low, medium, and high scoring groups for coach vote. *Indicates significant difference *p* < 0.05 between sub-groups. MTC, Mental Toughness Coach; SSG, Small sided games.

The sub-discipline performance test scores for participants in the categorized groups for disposal efficiency and coach vote are reported in Figures 2, 3 respectively, with descriptive values provided at Table 4. For disposal efficiency (Figure 2), when examining group differences between categories in regards to sub-discipline tests scores, a significant difference was only found overall for SSG test score [*F*_(2,18)_ = 4.893, *p* = 0.20]. Bonferroni post hoc comparisons indicated that there was a significant difference (*p* = 0.019, *d* = 2.2) between the high performing group (*M* = 5.53, *SD* = 0.31) and the low performing group (*M* = 5.01, *SD* = 0.14). For coach vote (Figure 3), when examining group differences between categories in regards to sub-discipline tests scores, a significant difference was only found overall for the 3 × 1 km test score [*X*^2^(2) = 7.112, *p* = 0.029]. Pairwise comparisons indicated that there was a significant difference (*p* = 0.031, *d* = 1.62) between the high performing group (*M* = 630, *SD* = 8.51 s) and the low performing group (*M* = 665, *SD* = 29.31 s).

**Table 4:** Mean sub-discipline scores for participants who completed all tests.

**All**		**Disposal efficiency**			**Coach vote**		
***n*** **=** **21**		**Low (<60%) *n* = 4**	**Medium (60–70%) *n* = 10**	**High (> 70%) *n* = 7**	**Low (<1) *n* = 7**	**Medium (1–2) *n* = 8**	**High (> 2) *n* = 6**
Age	21.24 ± 3.11	19.50 ± 1.30	20.90 ± 2.33	22.71 ± 4.31	19.57 ± 1.72	22.13 ± 3.48	22.00 ± 3.52
3 × 1 km (s)	645.24 ± 26.28	647.25 ± 23.61	656.00 ± 30.41	628.71 ± 11.00	664.57 ± 29.31	639.75± 24.02	630 ± 8.51
MTC	43.19 ± 5.59	38.25 ± 7.81	44.20 ± 4.85	44.57 ± 4.28	40.29 ± 6.10	44.50 ± 3.90	44.83 ± 6.43
SSG Score	5.31 ±. 32	5.01 ±.014	5.28 ± 0.27	5.53 ± 0.31	5.16 ± 0.25	5.39 ± 0.38	5.40 ± 0.30
Disposal efficiency	67.33 ± 6.47	–	–	–	–	–	–
Coach vote	1.50 ± 1.14	–	–	–	–	–	–

*MTC, Mental Toughness Coach; SSG, Small Sided Games*.

To illustrate individual differences, we have chosen to model three different participants who scored at high, medium, and low levels for the SSG test. The predictive equation generated from the multivariate (interdisciplinary) model for disposal efficiency, which incorporates participant 3 × 1 km time, MTC score and SSG test score was used resulting in the following predictions:

1. Participant one (high SSG score) recorded the following scores; 3 × 1 km time = 664 s, MTC score = 46 and SSG = 5.72, with a predicted disposal efficiency of 70%.
2. Participant two (medium SSG score) recorded the following scores; 3 × 1 km time = 625 s, MTC score = 50 and SSG = 5.24, with a predicted disposal efficiency of 68%.
3. Participant three (low SSG score) recorded the following scores; 3 × 1 km time = 616 s, MTC score = 49 and SSG = 5.08, with a predicted disposal efficiency of 66%.

### Representative Task Design

The impact of the level of task representation of performance tests on outcome measures is best illustrated with an example again using the predictive equation for disposal efficiency generated from our multivariate GEE (Table 3). We compared the effect of an increase in the SSG test (high task representation) score with a comparative increase in the MTC (low task representation) score. If we revisit the above example of using individual participant scores, the participant's predicted mean disposal efficiency is 66%. If the participant increases their SSG (high task representation) score by 10%, from 5.31 to 5.84, this would increase their disposal efficiency to 72.2%. If the participant increases their MTC (low task representation) score by 10%, from 43.19 to 47.51, this would increase their disposal efficiency to 67.3%. In this case, the participant results in the performance test with the higher level of task representation has a greater bearing on the overall outcome variable of disposal efficiency than the performance test with the lower task representation.

In relation to the Goodness of Fit analysis, the SSG test (high task representation) has the lowest QIC value for both disposal efficiency and coach vote indicating the best model fit. The mental toughness rating (low task representation) had the highest QIC value for both disposal efficiency and coach vote indicating a relative poorer model fit.

## Discussion

This study set out to address the call for interdisciplinary research that could provide a more comprehensive understanding of sport performance. We compared whether monodisciplinary and interdisciplinary approaches related to individual constraints contribute to predict measures of match performance in terms of disposal efficiency and coaches' vote. This comparison was crucial to demonstrate that an interdisciplinary approach could better predict match performance than a monodisciplinary approach alone. In addition, our interdisciplinary approach was capable of quantifying individual differences and representative task design relative to competition performance. Collectively, this provides a comprehensive understanding of sport performance that is theoretically driven and has practical implications for athlete development.

Our study reconfirmed that a monodisciplinary approach is relevant to understand sport performance. Indeed, univariate (monodisciplinary) analyses showed that each physiological, psychological, and perceptual-cognitive-motor skill component of individual constraints predicted disposal efficiency. The perceptual-cognitive-motor skill component also on its own predicted coaches' vote. These findings are consistent with previous monodisciplinary studies in AF that have reported perceptual-cognitive-motor skill can predict; disposal efficiency (Piggott et al., 2019b), as well as talent identified and non-talent identified athletes (Woods et al., 2016a). Moreover, physiological measures such as 20 m sprint time have also been able to predict talent identified and non-talent identified Australian footballers (Woods et al., 2017). Beyond AF, monodisciplinary studies have also been used to discriminate between skill levels using a video-based decision-making test in soccer (Keller et al., 2018) and by using a reactive agility test in rugby league (Gabbett and Benton, 2007). Therefore, there is merit to conduct sport performance research that measures these components in a monodisciplinary approach.

The interdisciplinary (multivariate analysis) approach in our study revealed that psychological and perceptual-cognitive-motor components of individual constraints contributed to significantly predict disposal efficiency. This finding is consistent with coach perception that superior performance in sport is related to psychological and perceptual-cognitive-motor skills (Steel et al., 2014; Zaichkowsky and Peterson, 2018). A likely reason for this is that coping with psychological pressure and making accurate decisions allows the player to apply their physical capability efficiently (less energy cost) to dispose of the ball effectively. Although psychological and perceptual-cognitive-motor components were significant predictors, when combined with the physiological component, there was better prediction of disposal efficiency (QIC value, see Table 3), in comparison to a monodisciplinary approach (QIC value, see Table 1). This finding is consistent with our prediction in hypothesis one. This finding is also consistent with previous research into AF that has reported better classification of talent identified and non-talent identified athletes based upon combined physical, technical, and perceptual-cognitive components (Woods et al., 2016b). Accordingly, it needs to be considered that in our study, the physiological component contributed to the multivariate model to allow better prediction. This indicates that physiological capacity is indeed important, as a footballer needs to run or sprint with the ball, but psychological and perceptual-cognitive-motor skill make a greater contribution to disposal of the ball effectively to a teammate. These findings resonate with the concern raised in the literature regarding the predominant focus upon physical or physiological components to explain sport performance (Cardinale, 2017). Rather, our findings demonstrate that psychological and perceptual-cognitive-perceptual skills, interacting with physiological capacity, is crucial to comprehensively understand sport performance.

Significant differences were found between some sub-groups for disposal efficiency in SSG and coaches' vote for the 3 × 1 km trial. These comparisons, however, focus on mean data and are not capable of demonstrating individual differences in performance. The better interdisciplinary prediction of competition performance established an equation, which was used to identify individual differences in sport performance. For example, we reported how scores from physiological, psychological and perceptual-cognitive-motor individual component constraints contributed to calculation of three different participant disposal efficiency scores (i.e., 70, 68, and 66%). This is consistent with our prediction in hypothesis two. Accordingly, our findings provide fine-grained detail of how individual constraints contribute toward competition task constraint performance. An individual differences approach has been lacking in the sports science literature with researchers focusing more upon group level analyses that presents mean data, rather than considering within-group differences (Woods et al., 2019). The reasons for this could be; first, sports scientists may have been initially interested in determining upon which components groups of athletes could be differentiated from non-athletes, and second, these group differences provided a means of validating measurement instruments through performance discrimination. Our example highlights that individual differences profiles were aligned with constraints theory that predicts the skill goal can be achieved through interaction of individual, task and environmental constraints (Higgins, 1977; Newell, 1986). Accordingly, our findings are consistent with other studies that have used constraints theory to predict and report individual differences in relation to performance at a specific instance in time (Müller et al., 2015a) and improvement of performance due to practice (Chow et al., 2008). Therefore, our developed equation could quantify individual performance, which is underpinned by an interacting constraints theoretical framework.

In utilizing an interdisciplinary approach, our findings showed that degree of representative task design of a test or measure can influence the predicted competition performance measure. To this, the MTC rating was considered lower in representative task design because it is purely a measure that requires an evaluation of athletic performance. This is unlike a performance test such as SSG that is considered high in representative task design because it includes perceptual information and action responses that are closely related to the competition setting of AF. Our analyses indicated that a consistent increase of 10% on each test results in different predictions of competition performance. Here, the test with high representative task design or the task that closely represents the context of generalization (competition), results in an increased level of predicted performance. Whilst representative task design has been popular in the literature (e.g., Gorman and Maloney, 2016; Woods et al., 2019), we are unaware of studies that have compared the relative contributions of higher and lower representative tasks to predict competition performance. There are, however, studies that have reported performance changes across lower to higher representative tasks in line with what would be expected relative to competition (Pinder et al., 2011a; Gorman and Maloney, 2016). Accordingly, our findings are consistent with hypothesis three and the theoretical predictions of representative task design. It is important to point out that lower representative task design does not mean that the task or measure should not be used. Indeed, as mentioned earlier, both psychological and perceptual-cognitive-motor components contributed to predict match performance indicating they are both valuable indicators of *in-situ* performance. It simply needs to be considered that a MTC rating (on its own) can under estimate actual competition performance.

The results of this study have a direct practical application for AF coaches, specifically in the development of players. An example could be that the coaching staff conduct the three tests (3 × 1 km time trial, SSGs and MTC) at the start of pre-season training period. The coaching staff can use these results to identify player strengths and weaknesses, inform individual development plans for each player and set specific improvement goals for them to achieve. Using this approach, aspects of training can then be individually tailored to developing the player and resources can be allocated accordingly. For example, if it was identified from the SSG testing that a player needs to improve their decision-making component in order to improve their total score, then coaches can create specific decision-making drills for the player to undertake. The playing group can then be retested after a certain time to see if the goals have been achieved.

## Limitations and Conclusions

There are limitations of this study that need to be considered. First, a potential limitation of interdisciplinary research is that performance needs to be assessed on all sub-discipline measures. This requires an increased time commitment to the research project by the participants than in monodisciplinary studies. In addition, the pool of participants in skilled athlete populations is significantly smaller than in the lesser skilled population. Athletes are also susceptible to injury, which can limit their participation in tasks that require a significant physical or motor component. Therefore, taking into consideration these difficulties, our sample size was appropriate for measures conducted in the field setting (see Triolet et al., 2013; Müller et al., 2015b). Second, we focused upon two competition performance indicators. There are of course other competition indicators such as number of possessions or contested possessions. Future research is clearly needed to determine how interacting interdisciplinary individual constraints predict a broader range of competition performance indicators.

In summary, we found that an interdisciplinary approach, underpinned by a constraints theoretical framework, provides a more comprehensive understanding of sport performance. This provides support to a constraints theory explanation of sport performance, which could also be extended to interdisciplinary performance change over time due to practice or experience (learning). Our interdisciplinary approach also allowed individual differences and representative task design to be quantified. This is vital because athlete development is based upon an individual approach to test and train, which requires the use of suitable representative tests. Collectively, it is surprising that teams of sports science researchers have not frequently collaborated to more comprehensively understand performance. Although we earlier discussed obstacles to interdisciplinary research, multi-disciplinary research teams exist across sports science institutes, sports organizations, and universities around the world. Perhaps the findings from this study might stimulate opportunities for collaboration between sports scientists and academics, which can help coaches better prepare individual athletes in a holistic manner for competition.

## Data Availability Statement

The datasets for this article are not publicly available because of confidentiality reasons. Requests to access the datasets should be directed to Ben Piggott, benjamin.piggott@nd.edu.au.

## Ethics Statement

The studies involving human participants were reviewed and approved by Notre Dame Australia Human Research Ethics Committee. The patients/participants provided their written informed consent to participate in this study.

## Author Contributions

All authors listed have made a substantial, direct and intellectual contribution to the work, and approved it for publication.

### Conflict of Interest

The authors declare that the research was conducted in the absence of any commercial or financial relationships that could be construed as a potential conflict of interest.
